# Bronchial Responsiveness Is Related to Increased Exhaled NO (FE_NO_) in Non-Smokers and Decreased FE_NO_ in Smokers

**DOI:** 10.1371/journal.pone.0035725

**Published:** 2012-04-26

**Authors:** Andrei Malinovschi, Christer Janson, Marieann Högman, Giovanni Rolla, Kjell Torén, Dan Norbäck, Anna-Carin Olin

**Affiliations:** 1 Department of Medical Sciences: Clinical Physiology, Uppsala University, Uppsala, Sweden; 2 Asthma and Allergy Research Centre, Uppsala University, Uppsala, Sweden; 3 Department of Medical Sciences: Respiratory Medicine and Allergology, Uppsala University, Uppsala, Sweden; 4 Centre for Research and Development, Uppsala University/County Council of Gävleborg, Sweden; 5 Department of Allergy and Clinical Immunology, University of Turin and Mauriziano Hospital, Turin, Italy; 6 Department of Occupational and Environmental Medicine, Sahlgrenska University Hospital, Gothenburg, Sweden; 7 Department of Medical Sciences: Occupational and Environmental Medicine, Uppsala University, Uppsala, Sweden; Lovelace Respiratory Research Institute, United States of America

## Abstract

**Rationale:**

Both atopy and smoking are known to be associated with increased bronchial responsiveness. Fraction of nitric oxide (NO) in the exhaled air (FE_NO_), a marker of airways inflammation, is decreased by smoking and increased by atopy. NO has also a physiological bronchodilating and bronchoprotective role.

**Objectives:**

To investigate how the relation between FE_NO_ and bronchial responsiveness is modulated by atopy and smoking habits.

**Methods:**

Exhaled NO measurements and methacholine challenge were performed in 468 subjects from the random sample of three European Community Respiratory Health Survey II centers: Turin (Italy), Gothenburg and Uppsala (both Sweden). Atopy status was defined by using specific IgE measurements while smoking status was questionnaire-assessed.

**Main Results:**

Increased bronchial responsiveness was associated with increased FE_NO_ levels in non-smokers (p = 0.02) and decreased FE_NO_ levels in current smokers (p = 0.03). The negative association between bronchial responsiveness and FE_NO_ was seen only in the group smoking less <10 cigarettes/day (p = 0.008). Increased bronchial responsiveness was associated with increased FE_NO_ in atopic subjects (p = 0.04) while no significant association was found in non-atopic participants. The reported interaction between FE_NO_ and smoking and atopy, respectively were maintained after adjusting for possible confounders (p-values<0.05).

**Conclusions:**

The present study highlights the interactions of the relationship between FE_NO_ and bronchial responsiveness with smoking and atopy, suggesting different mechanisms behind atopy- and smoking-related increases of bronchial responsiveness.

## Introduction

Bronchial hyperresponsiveness is one of the hallmarks of asthma and measurement of bronchial responsiveness has been used clinically for over 30 years for asthma diagnosis and monitoring [1]. Exhaled nitric oxide has been introduced as a tool for asthma diagnosis in subjects with symptoms of asthma [2] and for the monitoring of asthma therapy [3]. Fraction of nitric oxide in the exhaled air (FE_NO_) is a non-invasive marker of steroid-sensitive inflammation in the airways [4]. NO has also known bronchodilating and bronchoprotective physiological roles [5]. Apart from asthma, bronchial responsiveness and FE_NO_ are also associated with other factors such as atopy and smoking. Atopy is related both to increased bronchial responsiveness [6] and increased FE_NO_ [7], while smoking is associated with increased bronchial responsiveness [8] and decreased FE_NO_ [9].

A positive correlation between bronchial responsiveness and FE_NO_ has been found among subjects with allergic asthma [10] and in population-based studies of adults [11], [12] and children [13]. In these studies, after stratification for atopy, the association between bronchial responsiveness and increased FE_NO_ was statistically significant only among atopic individuals [11], [13].

An interaction of bronchial responsiveness with smoking and atopy has been previously suggested in a Spanish population-based study [14] where current smoking was associated with increased bronchial responsiveness only in non-atopic subjects. On the other hand, FE_NO_ is reduced to the same extent by current smoking in non-atopics and atopics [15]. This suggests that the association between FE_NO_ and bronchial responsiveness is affected both by smoking and atopy. No previous studies have analyzed how smoking and smoking amount influences the relationship between bronchial responsiveness and FE_NO_.

The aim of the present study was to investigate the association between bronchial responsiveness and FE_NO_, with special regard to how this association is influenced by smoking, smoking amount and atopy.

## Methods

### Ethics Statement

Written informed consent was obtained from each subject before inclusion in the study. The protocol was approved by the Uppsala Ethics Committee (decision 131/1999 for Swedish multicentre application for Uppsala and Gothenburg) and Verona Ethics Committee (decision 74/1998 for Italian multicentre ECHRS II application including Turin).

### Study participants

The European Community Respiratory Health Survey (ECRHS) is an international multicenter study of asthma and allergy. The first part, ECRHS I, was conducted in 1990–4 and the follow-up study, ECRHS II, in 1999–2001. The design of ECRHS I and II has been published in detail [16], [17].

The present study included 468 subjects from the random sample of three of ECRHS II centers, Gothenburg (n = 225) and Uppsala (n = 175) (both Sweden) and Turin (n = 68) (Italy), who have undergone stage 2 of ECRHS I and in ECRHS II have answered the main questionnaire, performed measurements of FE_NO_, lung function tests and methacholine challenge. No subjects on daily inhaled steroids and/or oral antileukotrienes were included in the present analyses. Details regarding the selection of the subjects in these three centers are available in another publication [18].

### Methacholine challenge

Methacholine challenge was carried out using a dosimeter (Mefar, Brescia, Italy). Methacholine challenge dose-response slope (“slope”) was calculated as the regression coefficient of percentage decline in FEV_1_ on log dose of methacholine and then reciprocally transformed to satisfy statistical assumptions of multiple regression [19]. Its values range from 1 to 20. Two units of change in “slope” corresponds to one unit of change in log_10_(PD_20_), or 3.32 doubling doses [20]. This relationship has been used to express the results in doubling doses in the manuscript. After transformation a low "slope”, like low PD_20_, was indicative of increased bronchial responsiveness. All subjects were instructed to refrain from smoking for at least 1 hour before lung function and methacholine reactivity measurements.

### Exhaled NO

Exhaled NO measurements were done according to ATS/ERS recommendations [21]. Exhaled NO measurements were carried out on a different day than methacholine challenge. Different techniques and flow rates of measuring FE_NO_ were used in different centers - offline measurements at 350 mL s^−1^ in Turin and online measurements at 50 mL s^−1^ in Uppsala and Gothenburg. The methods are described in more detail in another publication [18]. All subjects were instructed to refrain from smoking for at least two hours before measurements of exhaled NO, in order to exclude any potential confounding effects of acute smoking.

### Smoking habits, atopy and asthma diagnosis

**Smoking habits** were questionnaire-assessed. A subject was considered as being a current smoker if he/she had been smoking for more than one year or at least 20 packs of cigarettes and was still smoking the month before the study. The number of smoked cigarettes per day and cigarette consumption in pack-years was also questionnaire-assessed.

**Specific IgE** was measured against *Dermatophagoides pteronyssinus*, cat, timothy grass and *Cladosporium herbarum*, using the Pharmacia CAP System (Pharmacia Diagnostics, Uppsala, Sweden). A person was defined as **atopic** if the titers against at least one of the tested allergens were ≥0.35 kU/L.

**Current asthma** diagnosis was defined having self-reported physician-diagnosis of asthma and at least one asthma symptom or taking regular antiasthmatic medication during the last 12 months preceding the study.

### Lung function

Forced expiratory volume in one second (FEV_1_) was measured with a standardized method with different spirometers in different study centers, as previously described [18]. FEV_1_ was expressed as % of the predicted value [22].

### Statistics

Statistical analyses were performed using STATA 8.0 software (Stata Corp., 2001, Texas, USA). Different FE_NO_ measurement techniques [23], NO analysers [24] and exhalation flow rates were used and we therefore divided FE_NO_ in quartiles for each centre and pooled the data for the three centers instead of analyzing the absolute values of FE_NO_ for each centre.

Trend tests were applied when analyzing the association between FE_NO_ quartiles and other variables (Table 1). Simple linear regressions between slope values and FE_NO_ quartiles were performed. Interactions with smoking and atopy were studied in multiple linear regression models where adjustments were made for factors known, from literature, to affect bronchial responsiveness and FE_NO_. The interactions were also tested by a meta-analysis of corresponding multiple regression linear models when using absolute value of FE_NO_ instead of FE_NO_ quartiles for the respective three study centers. Heterogeneity between centers regarding the interaction of smoking respectively atopy with the relation between FE_NO_ and bronchial responsiveness was tested by means of a meta-analysis of the three centers. A p-value of <0.05 was considered statistically significant.

**Table 1:** Descriptive table of subjects divided according to their FE_NO_ levels (n (%) or arithmetic mean ± SD or arithmetic mean (95%CI)).

	FE_NO_ Q_1_ (n = 115)	FE_NO_ Q_2_ (n = 118)	FE_NO_ Q_3_ (n = 117)	FE_NO_ Q_4_ (n = 118)	p-value
***Slope*** †	7.86±2.16	7.78±1.80	7.91±1.63	7.51±2.06	0.25
***Atopy*** ‡	20 (18.5%)	30 (26.5%)	36 (31.9%)	45 (40.9%)	<0.001
***Current smoking*** §	37 (32.5%)	21 (18.1%)	15 (12.9%)	11 (9.5%)	<0.001
***Cigarettes/day***	14 (11, 17)	11 (6, 15)	8 (4, 12)	6 (2, 11)	0.002
***Pack-years***	22 (18, 26)	16 (10, 23)	16 (11, 20)	11 (3, 19)	0.003
***Male gender***	45 (39.1%)	58 (49.1%)	72 (61.5%)	76 (64.4%)	<0.001
***Height (cm)***	169.3±8.4	172.8±9.1	174.8±10.5	175.3±11.0	<0.001
***Weight (kg)***	74.0±15.2	76.9±14.8	77.5±14.2	78.2±15.2	0.03
***BMI (kg/m^2^)***	25.7±4.23	25.6±3.73	25.3±3.53	25.3±3.65	0.36
***Age (years)***	43.2±7.59	43.2±7.43	43.8±7.10	42.2±6.81	0.46
***Current asthma*** #	5 (4.4%)	4 (3.5%)	4 (3.4%)	13 (11.2%)	0.03
***FEV_1_ (%pred)***	105±13	107±14	110±13	107±13	0.22

All the given p-values are for trends across FE_NO_ quartiles.

† Methacholine challenge dose-response slope.

‡ Information on atopy status was missing in 24 patients.

§ Information regarding smoking habits was missing in 6 patients.

# Information regarding current asthma was lacking in 6 patients.

## Results

The characteristics of the study population are presented in Table 1. Subjects with higher FE_NO_ levels were characterized by a higher prevalence of atopy and lower prevalence of current smoking, whereas no significant association was found between FE_NO_ and slope values. Male gender, current asthma as well as increased height and weight, were associated with increased FE_NO_ levels.

### Selection bias – participants vs. non-participants

Participants who performed FE_NO_ measurements were more likely to be men (50 vs. 44%, p = 0.02) and had a slightly higher mean age (43.2±0.3 vs. 41.2±0.3 years, p<0.0001) than participants who did not undergo FE_NO_ measurements. No significant differences were found concerning bronchial responsiveness, smoking habits, atopy, physician diagnosed asthma, current asthma or body mass index between subjects who performed FE_NO_ measurements and subjects who did not.

### Effects of atopy and smoking on FE_NO_

**Figure 1:**
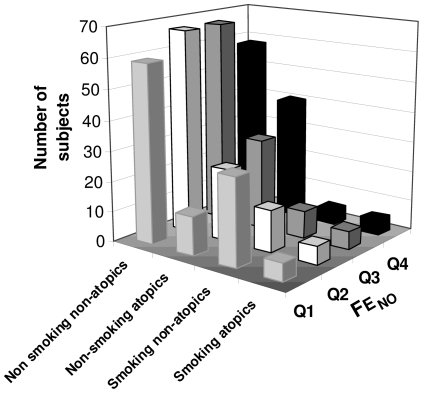
Number of subjects in each FE_NO_ quartile (FE_NO_ Q_1_–Q_4_) for non-smoking non-atopics, non-smoking atopics, smoking non-atopics and smoking atopics, respectively.

Dividing the subjects after current smoking and atopy status (information available in 438 subjects), we obtained four groups: non-smoking non-atopic (n = 251), non-smoking atopic (n = 107), smoking non-atopic (n = 57) and smoking atopic subjects (n = 23). Comparing the distribution of subjects into different FE_NO_ quartiles in the above mentioned groups, the group of non-smoking non-atopic subjects had lower FE_NO_ levels than the group of non-smoking atopic subjects (p = 0.01) and higher values than the smoking non-atopic subjects (p<0.001) (Figure 1). No differences in FE_NO_ were found between non-smoking non-atopics and the smoking atopic subjects (p = 0.96).

### Effects of smoking status on the relationship between bronchial responsiveness and FE_NO_

**Figure 2:**
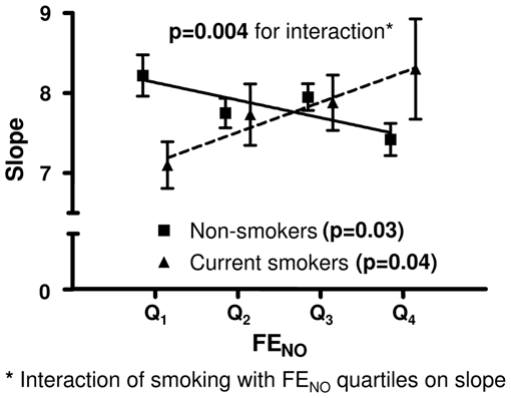
Methacholine challenge dose-response slope for all subjects divided upon their FE_NO_ quartiles values and smoking status. Data is presented as mean values ± standard error of the mean and a regression line (p-value in the brackets) is drawn for non- and current smokers, respectively.

Among non-smokers increased bronchial responsiveness was associated with increased FE_NO_ values while an opposite trend was seen among current smokers (Figure 2). There was a statistically significant difference in the association between slope and FE_NO_ in non- and current smokers (p-value for interaction = 0.004).

In Table 2 the results are expressed as doubling doses of methacholine. The interaction between smoking and FE_NO_ in relation to bronchial responsiveness remained significant after adjusting for gender, study centre, FEV_1_(%pred), age, height, weight, atopy, current asthma (Table 2). When stratifying for atopy, a significant interaction of smoking status with FE_NO_ quartiles on airway responsiveness was found only among atopic subjects (Table 2). No heterogeneity was found between centers regarding the interaction of current smoking with the association bronchial responsiveness and FE_NO_ (p = 0.60). Significant trends for increasing bronchial responsiveness with increasing FE_NO_ levels were found in all subjects (p = 0.009) and atopic subjects (p = 0.004) when a subanalysis was performed in Uppsala and Gothenburg centers. Moreover, the interactions with smoking remained statistically significant for all subjects (p = 0.012) and atopic subjects (p = 0.018).

**Table 2:** The difference (Δ) in bronchial responsiveness (BR), expressed as doubling doses of methacholine§, between the first FE_NO_ quartile (Q_1_) and the other quartiles (Q_2_–Q_4_) in all subjects, atopics and non-atopics, after stratifying for smoking.

	Difference in BR	Non-smokers	Current smokers	p_interaction_
**All subjects (n = 432)**	**ΔQ_1_–Q_2_**	0.83	0.08	0.011#
	**ΔQ_1_–Q_3_**	1.00	−0.91	
	**ΔQ_1_–Q_4_**	1.29	−1.23	
	**p_trend_** *	0.015	0.17	
**Atopics (n = 128)**	**ΔQ_1_–Q_2_**	1.58	−0.28	0.008
	**ΔQ_1_–Q_3_**	2.46	−1.39	
	**ΔQ_1_–Q_4_**	3.68	−3.87	
	**p_trend_** *	<0.001	0.11	
**Non-atopics (n = 304)**	**ΔQ_1_–Q_2_**	0.68	0.02	0.22
	**ΔQ_1_–Q_3_**	0.63	−1.23	
	**ΔQ_1_–Q_4_**	0.35	−0.88	
	**p_trend_** *	0.60	0.31	

§ *Slope was the outcome of the regression model and doubling doses were obtained by multiplying the regression coefficients with 1.66, as described in the* *Methods* *.*

* *p-value for trend represents the statistical significance for the association between bronchial responsiveness and FE_NO_ quartile (used as a qualitative variable).*

# p-value *for interaction represents the significance of interaction of smoking status with FE_NO_ quartile on airways responsiveness.*

*All the coefficients and p-values are adjusted for gender, study centre, FEV_1_(%pred), age, height, weight, atopy, current asthma.*

The interaction between smoking and FE_NO_ in relation to bronchial responsiveness was also found when FE_NO_ was expressed as absolute FE_NO_ values (p = 0.01).

The number of smoked cigarettes was correlated negatively to slope (p = 0.003) and current smokers who showed the lowest quartile of FE_NO_ were those who were smoking more cigarettes and had a higher pack-years consumption (p = 0.002 and p = 0.003, see Table 1). Nevertheless, the cigarette consumption in pack-years was not significantly related to slope (p = 0.18).

**Figure 3:**
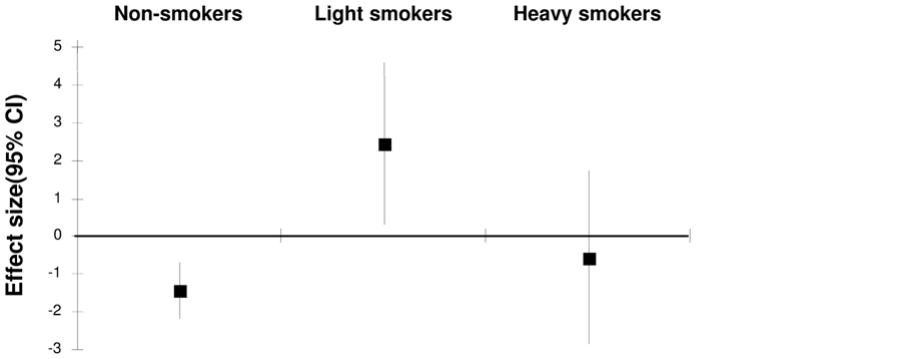
Effect size* (95%CI) for the association between slope and FE_NO_ (log-transformed) in non-, light (<10 cigarettes/day) and heavy smokers (≥10 cigarettes/day). * The effect size is the regression coefficient obtained by linear regression models with slope as outcome and log-transformed FE_NO_ as dependent variable where the estimates of the three centres were combined by meta-analysis.

Dividing current smokers into two groups, a positive association between slope and FE_NO_ quartile could be seen only in the group smoking less <10 cigarettes/day (p = 0.008) and not in the group smoking ≥10 cigarettes/day (p = 0.81) (Figure 3). These relations were consistent after adjusting for pack-years consumption, and also after additional adjustments for age, gender, height, weight, lung function, current asthma, atopy, study centre (p = 0.03). Performing in such a model a test of interaction of “light”/“heavy” smoking with FE_NO_ quartile on bronchial responsiveness a trend towards a significant interaction was found (p = 0.055). The positive association between slope and absolute levels of FE_NO_ could be found in subjects smoking less than 10 cigarettes/day in Gothenburg and less than 13 cigarettes/day in Uppsala (both p<0.05) (Table S1).

### Effect of atopy on the relationship between bronchial responsiveness and FE_NO_

**Figure 4:**
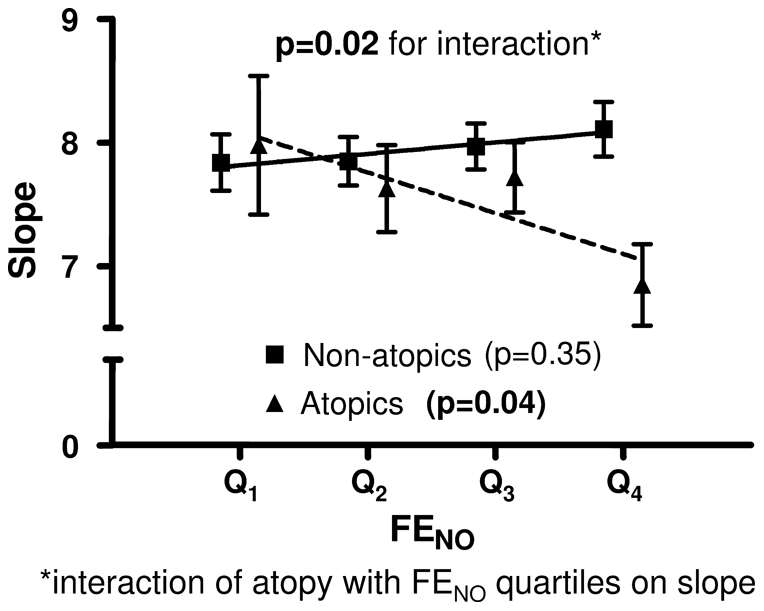
Methacholine challenge dose-response slope for all subjects divided upon their FE_NO_ quartiles values and atopy status. Data is presented as mean values ± standard error of the mean and a regression line (p-value in the brackets) is drawn for non-atopics and atopics, respectively.

A positive correlation was found between increased bronchial responsiveness (decrease of slope) and increased FE_NO_ among the atopic subjects whereas no significant correlation was found among the non-atopics (Figure 4, Table 3). The difference in association between bronchial responsiveness and FE_NO_ in atopics and non-atopics was statistically significant and the interaction of atopy with FE_NO_ quartiles on bronchial responsiveness remained statistically significant after adjusting for gender, study centre, FEV_1_(%pred), age, height, weight, atopy, current asthma (Table 3). No significant heterogeneity between centers was found regarding the interaction of atopy with the association between slope and FE_NO_ (p = 0.13).

**Table 3:** The difference (Δ) in bronchial responsiveness (BR), expressed as doubling doses of methacholine§, between the first FE_NO_ quartile (Q_1_) and the other quartiles (Q_2_–Q_4_) in all subjects, non-smokers and current smokers, after stratifying for atopy.

	Difference in BR	Non-atopics	Atopics	p_interaction_ #
**All subjects (n = 432)**	**ΔQ_1_–Q_2_**	0.46	0.91	0.012
	**ΔQ_1_–Q_3_**	0.27	1.64	
	**ΔQ_1_–Q_4_**	0.10	2.46	
	**p_trend_** *	0.91	0.006	
**Non-smokers (n = 352)**	**ΔQ_1_–Q_2_**	0.68	1.58	0.004
	**ΔQ_1_–Q_3_**	0.63	2.46	
	**ΔQ_1_–Q_4_**	0.35	3.68	
	**p_trend_** *	0.60	<0.001	
**Current smokers (n = 80)**	**ΔQ_1_–Q_2_**	0.02	−0.28	0.71
	**ΔQ_1_–Q_3_**	−1.23	−1.39	
	**ΔQ_1_–Q_4_**	−0.88	−3.87	
	**p_trend_** *	0.31	0.11	

§ *Slope was the outcome of the regression model and doubling doses were obtained by multiplying the regression coefficients with 1.66, as described in the* *Methods* *.*

* *p-value for trend represents the statistical significance for the association between bronchial responsiveness and FE_NO_ quartile (used as a qualitative variable).*

# p-value *for interaction represents the significance of interaction of atopy status with FE_NO_ quartile on airways responsiveness.*

*All the coefficients and p-values are adjusted for gender, study centre, FEV_1_(%pred), age, height, weight, atopy, current asthma.*

Dividing the participants into non-smokers and smokers the relationship between bronchial hyperresponsiveness and FE_NO_ was found to be significant only among non-smoking subjects (Table 3).

Significant trends for increasing bronchial responsiveness with increasing FE_NO_ levels were found in all atopic subjects (p = 0.033) and all atopic, non-smoking subjects (p = 0.004) when a subanalysis was performed in Uppsala and Gothenburg centers. Moreover, the interactions with atopy remained statistically significant for atopic subjects (p = 0.04).

The interaction of atopy with the relationship between slope and FE_NO_ was also found to be significant when using absolute FE_NO_ values (p = 0.01).

### Three-way interaction between atopy, smoking and FE_NO_ on bronchial responsiveness

In a model where bronchial responsiveness was the outcome and three-way interactions between atopy, smoking and FE_NO_ were tested, only the interaction between atopy with FE_NO_ on bronchial responsiveness was significant (p = 0.005). This was consistent after adjusting for gender, study centre, FEV_1_(%pred), age, height, weight, atopy, current asthma (p = 0.003). The three-way interaction of smoking with atopy with FE_NO_ on bronchial responsiveness was not significant in unadjusted (p = 0.15) or adjusted model (p = 0.12).

## Discussion

The main finding of the present study is that bronchial responsiveness is associated with increased FE_NO_ levels in non-smokers and with decreased FE_NO_ levels in current smokers. Actually the inverse relationship between FE_NO_ and bronchial responsiveness was significant only in “light” smokers, suggesting possible different mechanisms of bronchial responsiveness in “light” and “heavy” smokers. Increased bronchial responsiveness was associated with increased FE_NO_ in atopic subjects while no such relationship could be seen in non-atopics. The nature of the interactions on the relationship between FE_NO_ and bronchial responsiveness with smoking and atopy appears to be even more complex, since the interaction with smoking was seen only in atopics, while the interaction with atopy was seen only in non-smokers.

We think there are many reasons why the inverse relationship between FE_NO_ and bronchial responsiveness in smokers cannot be explained simply by considering the negative effect of smoking on FE_NO_, on one hand, and the favoring effect of smoking on bronchial hyperesponsiveness [25], on the other hand, without a causal relationship between the two effects. First, constitutively produced NO may play a bronchoprotective role, which should be lost in smokers, due to a lower NOS-production of NO [26], [27] or an increased catabolism of NO [28], [29]. Evidence for a bronchoprotective role of NO exist both in experimental animal studies [30] [31], but also in human studies performed in asthmatic subjects [32] [33] where administration of different non-selective iNOS inhibitors resulted in increased bronchial responsiveness. Another possible explanation could be related to the smoking-induced neutrophil inflammation. Sputum neutrophils count has been found to be negatively correlated to FE_NO_ in smokers [34] and activation of neutrophils, *in vitro*, has been shown [35] to decrease NO, due to generation of peroxynitrite. Increased IL-16 has been linked with the neutrophilic inflammation [36], and IL-16 has been demonstrated to be increased in the airways of cigarette smokers, independent on the intensity of smoking [37]. Epithelial and subepithelial IL-16 immunoreactivity has been associated with increased bronchial responsiveness in humans with allergic asthma [38] and in an animal model of allergic asthma [39].

Moreover, in our study, decreased FE_NO_ was associated with increased bronchial responsiveness only in “light” smokers, in whom the bronchoprotective effect of NO may be particularly valuable. Structural changes of small airways are related to smoking amount [40] and thus, in “heavy” smokers, bronchial hyperresponsiveness is best explained by structural changes of small airways and lung parenchyma [41]. However, we acknowledge the limitation that the different effects of “light” and “heavy” smoking on the association between FE_NO_ and bronchial responsiveness could not be fully confirmed when performing a statistical interaction test (p = 0.055).

We were able to confirm in this large population sample that the previous reported association between FE_NO_ and increased bronchial responsiveness in adults [11]–[13] was significant only in atopic subjects. Atopy-related increase in FE_NO_ is due probably to the eosinophilic subclinical inflammation in the airways [42], as the link between FE_NO_ and eosinophilic inflammation is well known [43]–[45]. The mechanism behind increased bronchial responsiveness in atopic subjects is most probably due to a combination of subclinical eosinophilic inflammation and remodeling changes described in the airways of atopic subjects [46]. A Th_2_-driven allergic response via IL-4-IL-13 cytokines could well result in both increased NO [47], [48] and increased bronchial responsiveness [48], [49].

The present study fills a gap regarding the effect of smoking on the association between bronchial responsiveness and FE_NO_ and it also made it possible to analyze the interactions of atopy and smoking on the association between bronchial responsiveness and FE_NO_. The only group where we did find an association of increased FE_NO_ values with increased bronchial responsiveness was the group of non-smoking atopic individuals. We found similar levels of FE_NO_ among the non-atopic non-smoking subjects and atopic smoking subjects due to the fact that FE_NO_ is affected both by smoking and atopy.

The main weakness of the present study resides in the different methods to measure FE_NO_ in the participating centers. We used quartiles of FE_NO_ instead of absolute values of FE_NO_ and no heterogeneity between centers was found regarding the interaction of smoking and atopy, respectively, with the relationship between FE_NO_ and bronchial responsiveness. An indirect validation of this method of using FE_NO_ quartiles in the present material is obtained by confirming the previous results on the relationship between FE_NO_ and bronchial responsiveness [11]–[13]. The fact that in one center (Turin) FE_NO_ was measured by higher flow-rate, which theoretically can sample to a slightly higher extent the peripheral airways, appears to be scarcely influent in this study, as atopy does not affect alveolar NO [50] and current smoking leads only to minor decrease of alveolar in comparison with bronchial contribution to exhaled NO [51]. Moreover, the main results could be confirmed in a subanalysis performed only in Gothenburg and Uppsala. In our population sample atopic subjects are underrepresented in the current smokers group, probably because the subjects with atopy and bronchial hyperresponsiveness might be less prone to start smoking. However this does not appear to confound our results, since the proportion of atopics increase with each FE_NO_ quartile among the smokers without any corresponding increase in BR levels. COPD pathology is unlikely to have affected the results of the present study, as no subjects have a known COPD-diagnosis and only three subjects among the current smokers had a FEV1/FVC-ratio <0.70.

The difference in the relationships between bronchial responsiveness and exhaled NO in smokers and atopics respectively suggests that atopy- and smoking cause bronchial hyperresponsiveness through different pathophysiological mechanisms. The nature of the interactions between bronchial responsiveness and exhaled NO is complex as the interaction with smoking could be seen only in atopics while the interaction with atopy could be seen only in non-smokers. Further studies are needed in order to understand the mechanisms explaining how smoking and atopy influence the relationship between bronchial responsiveness and exhaled NO.

## Supporting Information

Table S1 The relation (beta coefficient from multiple linear regression models) between bronchial responsiveness (expressed as methacholine doubling dose) and FE_NO_ in smoking subjects in Uppsala and Gothenburg centers ^#^ after dividing them for current cigarette consumption with different arbitrary cut-off levels. All the coefficients and p-values are adjusted for gender, FEV_1_(%pred), age, height, weight, atopy, current asthma.
(DOCX)
