# Supplementary material for: Bronchial Responsiveness Is Related to Increased Exhaled NO (FENO) in Non-Smokers and Decreased FENO in Smokers
Source: PLoS One. 2012 Apr 26;7(4):e35725. doi: 10.1371/journal.pone.0035725 (PMC3338521; doi:10.1371/journal.pone.0035725)
Supplement: Table S1 — The relation (beta coefficient from multiple linear regression models) between bronchial responsiveness (expressed as methacholine doubling dose) and FENO in smoking subjects in Uppsala and Gothenburg centers # after dividing them for current cigarette consumption with different arbitrary cut-off levels. All the coefficients and p-values are adjusted for gender, FEV1(%pred), age, height, weight, atopy, current asthma. (DOCX) [file pone.0035725.s001.docx]

**Supporting Table 1.** The relation (beta coefficient from multiple linear regression models) between bronchial responsiveness (expressed as methacholine doubling dose) and FE_NO_ in smoking subjects in Uppsala and Gothenburg centers ^#^ after dividing them for current cigarette consumption with different arbitrary cut-off levels. All the coefficients and p-values are adjusted for gender, FEV_1_(%pred), age, height, weight, atopy, current asthma.

| **Cut-off for number of daily smoked cigarettes** | **Gothenburg** | | **Uppsala** | |
| --- | --- | --- | --- | --- |
|  | **< cut-off** | **>=cut-off** | **<cut-off** | **>=cut-off** |
| **10** **cigarettes** | 0.25 (p=0.03) | -0.20 (p=0.32) | - * | -0.27 (p=0.16) |
|  | (n=20) | (n=25) | (n=8) | (n=16) |
| **13 cigarettes** | 0.20 (p=0.07) | -0.40 (p=0.06) | 0.53 (p=0.04) | -0.03 (p=0.59) |
|  | (n=25) | (n=20) | (n=14) | (n=10) |
| **15 cigarettes** | 0.20 (p=0.06) | -0.40 (p=0.06) | 0.53 (p=0.04) | -0.03 (p=0.59) |
|  | (n=26) | (n=19) | (n=14) | (n=10) |
| **20 cigarettes** | 0.15 (p=0.08) | -0.37 (p=0.10) | 0.07 (p=0.76) | - * |
|  | (n=33) | (n=12) | (n=20) | (n=4) |

^#^ data from Turin not presented as the number of subjects was too low in order to perform multiple linear regression analyses

* missing values when there were too few subjects for the multiple linear regression analyses
